# The Iranian version of geriatric anxiety inventory (GAI-P): a validation study

**DOI:** 10.1186/s12955-019-1176-z

**Published:** 2019-07-11

**Authors:** Razieh Bandari, Majideh Heravi-Karimooi, Mojgan Miremadi, Leila Mohebbi, Ali Montazeri

**Affiliations:** 1 Social Determinants of Health Research Center, Semnan University of Medical Sciences, Semnan, Iran; 2 Elderly Care Research Center, College of Nursing & Midwifery, Shahed University, Tehran, Iran; 3 Faculty of Nursing Midwifery, Tehran University of Medical Sciences, Tehran, Iran; 4 Department of Health, Dezful University of Medical Sciences, Dezful, Iran; 5 Population Health Group Health Metrics Research Centre, Iranian Institute for Health Sciences Research, ACECR, Tehran, Iran; 6 Faculty of Humanity Sciences, University of Science & Culture ACECR, Tehran, Iran

**Keywords:** Anxiety, Elderly, Translation, Validity, Reliability, Geriatric anxiety inventory

## Abstract

**Background:**

Anxiety is one of the most common mental health problems experienced by the elderly that affects quality of life. This study aimed to assess the psychometric properties of the Persian version of the Geriatric Anxiety Inventory (GAI-P) in order to provide a valid instrument for measuring anxiety in this population.

**Methods:**

Forward-backward translation was used to translate the Geriatric Anxiety Inventory from English into Persian and was tested by 10 elderly to assess its face validity. Then a sample of elderly people attending health centers in Dezful, Iran completed the questionnaire. Validity was assessed using both exploratory and confirmatory factors analysis, known-groups comparison (abused and non-abused elderly) also was administered. The internal consistency of the instrument was assessed using Kuder-Richardson 20 coefficients (KR-20). Afterwards, the reliability and validity through assessing the correlation between the Persian version of the GAI-P and the SF-36, were measured.

**Results:**

In all 720 elderly completed the questionnaire. Of these data from 420 elderly were used for exploratory factor analysis and the data from the remaining 300 elderly were used for confirmatory factor analysis. The exploratory factor analysis showed a three-factor solution (cognitive, arousal and somatic) for the questionnaire that jointly explained 59.48% of the overall variance observed. The confirmatory factor analysis supported the three-factor solution and the second-order latent factor model. The findings indicated a positive and significant correlation between the two measures lending support to its concurrent validity (r = 0.67, *p* < .001).Ultimately, the Geriatric Anxiety Inventory was found to have a favorable internal consistency.

**Conclusion:**

This study confirmed that the GAI-P is a valid measure of anxiety in elderly population and now can be used in geriatric studies in Iran.

## Background

Evidence suggests that anxiety is one of the most common mental health problems experienced by the elderly [1]. Studies have shown that generalized anxiety affects 15 to 56% of adults in clinical settings [2] and is associated with depressive disorders and increased risk of mortality [3]. However, psychological disorders such as anxiety and dementia in the elderly are studied less than other types of disorders and accurate prevalence and incidence remain obscure [4]. This problem is in part due to methodological factors including diagnostic criteria for anxiety and the instruments that are usually used for measuring anxiety in the elderly [4]. Diagnostic difficulties and cognitive and psychosocial problems for measuring anxiety in elderly populations are well discussed elsewhere [5, 6]. Thus, careful screening of anxiety symptoms in elderly is the first step for identifying individuals who need further diagnostic measures and treatments [4]. It is argued that anxiety could severely affect quality of life in elderly population [7] and in turn low quality of life might influence physical, mental and social well-being further [8]. Good quality of life for older people can be defined as feeling better, doing the right thing in everyday life activities, and keep relationship with family and friends [9]. As such it seems that measuring anxiety in elderly is very relevant to quality of life.

Studies addressing determinants of quality of life in elderly generally focus on a limited number of domains, such as the presence of multi-morbidities [10, 11], visual impairment [12] and obesity [13], behavioral issues, such as higher levels of alcohol use [14, 15], smoking [16, 17] or active lifestyle [15, 18]. In addition to this, social factors have also been shown to influence quality of life in the ageing process. Examples of this include social and family relationships [19, 20] and socioeconomic status [21–23]. However, little research has been devoted to the analysis of the impact of mental disorders on quality of life in elderly.

At present, the prevalence of mental disorders in the elderly is increasing. The World Health Organization (WHO) estimates that nearly one out of every 10 elderly has anxiety [24]. Anxiety is characterized by widespread, indirect and unrealistic concerns about everyday life events or activities. Anxiety symptoms may occur on most days for at least six months. The most common symptoms include tachycardia, sleep problems, sweating, dizziness, gastrointestinal disorders, and nausea [25].

Although several instruments are used for measuring anxiety in elderly, very few have been designed specifically for this population [4]. As such the Geriatric Anxiety Inventory (GAI) was developed by Pachana et al. in order to measure anxiety symptoms in the elderly [4]. Since then the instrument was used by many investigators and is validated in many languages including Chinese, Spanish, French, Portuguese, Brazilian, Australian, Italian and Chilean [1, 26–34]. This study aimed to validate the instrument in Iran. Currently there is no such instrument available in Persian.

## Methods

### The geriatric anxiety inventory

The GAI is a 20-item questionnaire for measuring anxiety in older adults (adults aged 60 and over). The questionnaire has agree/disagree response categories and for estimating anxiety score the number of ‘agree’ responses are added giving a total score ranging from 0 to 20, with higher scores indicating higher levels of anxiety. The questionnaire consists of three dimensions including cognitive (11 items), arousal (5 items), and somatic (4 items). The GAI was developed and tested among samples of community-dwelling older adults and older adults receiving psychiatric services. Excellent internal consistency was observed in the original study for community-dwelling older adults (α = 0.92) and older adults receiving psychiatric services (α = 0.93) [4]. Moderate to strong correlations between the GAI and other anxiety measures provided an evidence for concurrent validity, with correlations ranging from 0.58 to 0.86 [4, 26, 35] However, relatively high correlations (r = 0.65–0.79) between the GAI and depression measures provided limited evidence for discriminant validity [35, 36].

### Translation procedure

After asking for permission the recommended process of forward-backward translation method was used to translate the questionnaire from English into Persian. Hence, first two experts who were fluent in both English and Persian translated the items to Persian. Then the two Persian translations were compared and mixed together to form a single forward version. The forward translation was re-translated into English by two other experts. Then, the backward English version was compared to the original questionnaire in order to insure that the main concepts were maintained. In order to verify content validity, a panel of experts evaluated the questionnaire. The panel was consisted of 5 experts on Persian language, gerontology, public health, health education and health psychology. They were asked to make necessary revisions in terms of grammar, using the right words and placing the items in the best order. Accordingly the experts made no changes to the questionnaire. Then, the questionnaire was pre-tested among 10 elderly in order to assess face validity. They were asked to indicate if they had any difficulty to complete the questionnaire. They were also asked if there were any ambiguous word or phrases and if items were relevant to themselves. Almost all elderly reported no difficulty in responding to questionnaire and found the questionnaire easy to understand and very relevant. Next, the provisional Persian version of the questionnaire was prepared and was subjected to psychometric evaluation.

### Participants and the study setting

The study was conducted in Dezful, Iran (located in south) from January to June 2017. The study included a sample of older adults attending health centers for routine visits. All participants were asked to complete the study questionnaires in a calm setting. In the case of illiterate individuals two of us (LM or MM) helped people to complete the questionnaires. In all instances completion of the questionnaires took 15 min. The following inclusion criteria were used: being 60 years or older, and willingness to participate in the study. Exclusion criteria included physical disability such as hearing or speech impairment, and mental and cognitive disorders. The cognitive disorder was indicated using the abbreviated mental test and based on suggested cut-off points those with score of less than 6 were excluded [37].

### Additional measures

1. The demographic characteristics of the participants including age, sex, marital status, Living condition education level, number of children, economic status, the risk of chronic diseases were asked and recorded. The self-reported economic status of the elderly indicated as poor, intermediate, and good.
2. For the purpose of discriminant analysis the Elderly Abuse in the Household was administered and completed by participants. The questionnaire contains 49 items tapping into eight subscales: caring neglect (11 items), psychological abuse (8 items), physical abuse (4items), financial abuse (6 items), curtailment of personal autonomy (10 items), abandonment (4 items), financial negligence (4 items), and emotional neglect (2 items). The response categories are ‘yes’, ‘no’ and ‘ not applicable’. The score range from 0 to 100 where the higher scores indicate higher levels of abuse. To calculate scores the following formula was used:

$$ \left(\mathrm{Number}\ \mathrm{of}\ \mathrm{yes}\ \mathrm{answers}/\left(\mathrm{total}\ \mathrm{items}\hbox{-} \mathrm{NA}\ \mathrm{items}\right)\right)\ast 100. $$

The psychometric properties of the instrument in Iran are well documented. Calculating the Cronbach’s alpha coefficient (0.90–0.97) and stability by test-retest (0.99) confirm that the questionnaire has the desired reliability [38]. The questionnaire was completed by participants.

3. The Persian version of SF-36 questionnaire (SF-36) was used for concurrent volatility [39]. It is a generic measure of quality of life and contains eight health dimensions including: physical functioning, role physical, bodily pain, general health, vitality, social functioning, role emotional, and mental health. The scores for eight dimensions range from zero to 100 where zero indicates worse and 100 indicates best conditions. The reliability and validity of the Iranian version of the questionnaire have already been confirmed [40].

### Statistical analysis

Several statistical analyses were performed to evaluate psychometric properties of the Iranian version of geriatric anxiety inventory as described below:

**Construct validity**: It was assessed using both the exploratory factor analysis (EFA) and confirmatory factor analysis (CFA) with two different samples (*n* = 420, and *n* = 300, respectively). The Kaiser–Meyer–Olkin (KMO) Index and Bartlett’s Test of sphericity were used to assess sampling adequacy; KMO > 0.8 denoted an adequate sample [41, 42]. The latent factors of the EFA were extracted by maximum likelihood using Promax rotation and a screen plot. The presence of an item in a factor was determined as approximately 0.2 using the equation CV = 5.152 ÷√ (n-2),where CV = the number of extractable factors and *n* = sample size [43]. According to the three-indicator rule, there must be at least three items for each latent variable in the EFA [44]. Items with communalities less than 0.5 were excluded from the EFA. The Confirmatory Factor Analysis (CFA) was conducted to evaluate the measurement model for the GAI considering 20 items and 3 factors that extracted according to previous EFA. Because of dichotomous indicators in measurement model of GAI, we used WLSMV *(Weighted Least Square Mean and Variance adjusted)* estimation method, which use the tetra-choric correlation matrix of indicators [45, 46]. Model fit was determined based on fit indices as follows: Comparative Fit Index (CFI), Tucker-Lewis Index (TLI), Root Mean Square Error of Approximation (RMSEA), Standardized Root Mean Square Residual (SRMR), and Weighted Root Mean Square Residual (WRMR) [47].

**Discriminant validity**: The known groups comparison was used to perform discriminant validity. It was hypothesized that the questionnaire should discriminate between abused and non-abused elderly. The total score and scores obtained for each of the three dimensions of the scale were compared between the two groups by using an independent sample t test.

**Concurrent validity**: The Pearson’s correlation coefficient was used to assess concurrent validity.

**Reliability**: Internal scale reliability was examined using the Kuder-Richardson Formula 20 (RK-20). This is similar to the Cronbach’s alpha for dichotomous scores. Values can range from 0.00 to 1.00 (sometimes expressed as 0 to 100) and it is often said that a high KR-20 coefficient (e.g., > 0.90) indicates that an instrument has desirable internal consistency.

## Results

### Characteristics of the study sample

In all 720 elderly took part in the study. Of these 372 (51.7%) were male, 69.5% (*n* = 501) were married, most were illiterate (58.3%), and 42.1% were housewives. The majority of participants were living with their spouse (69%) and 33% were classified as middle-income. The characteristics of the participants are shown in Table 1.

**Table 1:** The characteristics of study participants (*n* = 720)

	Number (%)
Gender	
Male	372 (51.7)
Female	348 (48.3)
Age group (year)	
≤7	393 (54.5)
71–80	229 (32.0)
> 80	98 (13.5)
Living condition	
Alone	45 (6.25)
With wife	518 (71.95)
With Children	154 (21.25)
Others	4 (0.55)
Education level	
Illiterate	421 (58.3)
Primary	216 (30.0)
Secondary	71 (10.0)
Higher	12 (1.7)
Marital status	
Married	501 (69.5)
Divorced	219 (30.5)
Economic Status	
Poor	233 (32.4)
Intermediate	240 (33.3)
Good	247 (34.3)
Number of children	
0	3 (0.5)
1–3	79 (11.0)
4–6	288 (40.0)
7–9	268 (37.1)
> 10	82 (11.4)
Employment status	
Housewife	303 (42.1)
Employed	69 (9.5)
Retired	161 (22.4)
Un employed	187 (26.0)
The risk of chronic diseases	
Yes	287 (41.2)
No	433 (58.8)

### Construct validity

**Fig. 1:**
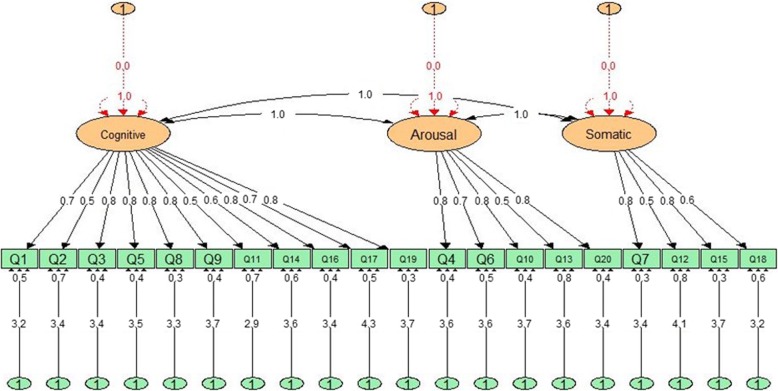
The results obtained from Confirmatory Factor Analysis (CAF) for the GAI-P

- Exploratory factor analysis: The Kaiser–Meyer–Olkin index (KMO) was 0.967 and Bartlett’s test (χ^2^ = 4.21, df = 190 (*p* < 0.001) indicated sampling adequacy. The EFA resulted in extraction of three factors *(cognitive, arousal and somatic related symptoms)* that jointly explained 59.5% of the total variance observed. The detailed results are presented in Table 2.
- Confirmatory factor analysis: Fig. 1 displays the 20 items as the indicators or observed variables and 3 extracted components as the factors or latent variables. All items had loading greater than 0.50 and the fit indices were as follows: χ^2^ = 290.9, DF = 167, CFI = 0.96, TLI = 0.96, RMSEA = 0.05 (95%CI = 0.04–0.06), SRMR = 0.04, and WRMR = 0.94. All values had acceptable thresholds and confirmed the hypothesized measurement model for the instrument.

**Table 2:** The results obtained from exploratory factor analysis for the GAI*

Item’s number	Items	Factor 1	Factor2	Factor3
1	I worry a lot of the time	**0.728**	0.226	0.322
2	I find it difficult to make a decision	**0.699**	0.120	0.165
3	I often feel jumpy	**0.726**	0.410	0.201
5	I often cannot enjoy things because of my worries	**0.741**	0.346	0.254
8	I think of myself as a worrier	**0.789**	0.491	0.411
9	I can’t help worrying about even trivial things	**0.651**	0.215	0.491
11	My own thoughts often make me anxious	**0.590**	0.202	0.122
14	I always anticipate the worst will happen	**0.662**	0.323	0.193
16	I think that my worries interfere with my life	**0.732**	0.105	0.215
17	My worries often overwhelm me	**0.724**	0.160	0.280
19	I miss out on things because I worry too much	**0.745**	0.216	0.359
4	I find it hard to relax	0.347	**0.747**	0.024
6	Little things bother me a lot	0.032	**0.715**	0.096
10	I often feel nervous	0.165	**0.753**	0.010
13	I think of myself as a nervous person	0.065	**0.556**	0.342
20	I often feel upset	0.002	**0.750**	0.229
7	I often feel like I have butterflies in my stomach	0.032	0.096	**0.773**
12	I get an upset stomach due to my worrying	0.065	0.324	**0.593**
15	I often feel shaky inside	0.208	0.193	**0.763**
18	I sometimes feel a great knot in my stomach	0.171	0.0.09	**0.700**

^*^Kaiser-Meyer-Olkin measure of sampling adequacy = 0.967, Bartlett’s test of Sphericity was significant (*p* < .001)

Factor1; cognitive, Factor2: arousal Factor3: somatic

### Discriminant validity

The results showed that the GAI well differentiated between abused and non-abused elderly as hypothesized. The mean score for the whole scale and its dimensions was significantly differed in both groups (*p* < 0.001) (Table 3).

**Table 3:** Comparison known groups: the mean score on the basis of an abuse of the Geriatric Anxiety Inventory (abuse and non abuse)

	Abused (*n* = 254)	Non-abused (*n* = 466)	
	Mean (SD)	Mean (SD)	P
Cognitive	34.44 (5.61)	32.08 (5.43)	.001
Arousal	18.75 (3.26)	17.57 (3.06)	.001
Somatic	12.77 (2.28)	11.83 (2.21)	.001
Total	65.81 (10.64)	61.52 (9.98)	.001

### Concurrent validity

To assess concurrent validity, the correlation between the GAI and the SF-36 was tested. The findings indicated a positive and significant correlation between the two measures lending support to its concurrent validity (r = 0 .67, *p* < .001). The results are shown in Table 4.

**Table 4:** The correlation between the GAI-P and SF-36

Variable	Cognitive	Arousal	Somatic
Physical function	.368^a^	.334^a^	.240^b^
Role physical	.153^b^	.130	.092
Bodily pain	.418^a^	.445^a^	.296^a^
General health	.093	.023	.092
Vitality	.479^a^	.443^a^	.270^a^
Social function	.357^a^	.362^a^	.205^b^
Role emotional	.673^a^	.578^a^	.535^a^
Mental health	.458^a^	.458^a^	.271^a^

^a^Correlation is significant at the 0.01 level

^b^Correlation is significant at the 0.05 level

### Reliability

Table 5 presents the results for internal consistency with Kuder-Richardson 20 coefficients (KR-20). The findings indicated that that all coefficients were above standard threshold for reliability.

**Table 5:** The Kuder-Richardson coefficients for the GAI

	Number of Items	Kuder-Richardson Coefficient
Cognitive (C)	11	0.916
Arousal (A)	5	0.852
Somatic (S)	4	0.779
Total	20	0.952

## Discussion

We translated the GAI into Persian and confirmed its validity and reliability. All recommended steps for translation were followed to ensure cultural agreement and symmetry for the instrument (43). The findings showed that the Persian version of Geriatric Anxiety Inventory (GAI) is a valid instrument and consistent with the original and the Chinese and Spanish validation studies is a three-factor instrument [1, 29]. However, the Canadian and Chilean version showed one-dimensionality [34, 48]. There is also a study that identifies a four-factor structure for the questionnaire when it was applied in a population of older Americans [49]. Molde et al. argued that different factor structures observed in several studies might be due to different reasons such as cultural issues, linguistic aspects, and sample characteristics [50].

Three distinct components were extracted for the instrument, that jointly accounted for 59.5% of the total variance observed. The finding was in agreement with the original instrument. As such cognitive, somatic and arousal factors seem to coincide with the measurement of anxiety in older adults and are important dimensions of anxiety [4]. The ‘cognitive and somatic’ factors appear to be rather coincident with those reported by previous studies measuring anxiety in older adult with the GAI and the ‘arousal’ also has been identified by previous studies as an important dimension of anxiety. It is relevant to note that most GAI items were found to load on the cognitive related factor, which includes items discussing on worry and presentiment. Worry is a clinical feature of anxiety to which elderly seem especially sensitive and, is the main feature of generalized anxiety disorder (GAD), one of the most prevalent anxiety disorders later in life [29, 51].

Internal consistency (Kuder-Richardson 20 coefficient) in this study was quite high and consistent with previous studies, such as the Spanish, Portuguese, Canadian, Brazilian Portuguese, Italian, Chinese and Australian versions [1, 4, 5, 7–9, 24]. For instance Márquez-González et al., also reported similar findings [29]. In a study by Yan et al. among elderly people living in Beijing community the Cronbach’s alpha coefficient was more than 0.94 [1]. Italian translation of the questionnaire showed a relatively lower coefficient (Cronbach’s α = 0.76) [32]. In a study by Gould et al. internal consistency with Kuder-Richardson 20 coefficients (KR-20) of 0.89 and 0.80 was reported [52]. The Brazilian Portuguese version of GAI (GAI-BR) showed high internal consistency (Cronbach’s α = 0.91) and strong and significant test-retest reliability (ICC = 0.85, *p* < 0.001) [33]. The Australian study among older Chinese immigrants reported good internal reliability (Cronbach’s *α* = 0.95) [53]. In addition, we found that the Persian GAI had a good level of content validity. The results showed a consistent semantic similarity between the GAI-P and its original English version. However, all these might point to the fact that Iranian elderly similar to other elderly people in other countries and cultures relatively have the same concerns and perhaps suffer from very similar challenges.

Using the Elderly Abuse in the Household it was found that about 35% of elderly were suffering from abuse and as expected the anxiety scores were higher in abused elderly as compared to the non-abused elderly. Evidence suggests that abuse of the elderly is one of the major causes of physical injury, illness, loss of efficiency, isolation [54], helplessness, sin, fear, embarrassment, post traumatic syndrome [55], anxiety, mental stress [56], despair, hopelessness, decrease Satisfaction with life, health and safety [57, 58], loss of property and also a threat to the survival and quality of life of the victims [54, 59].

### Application

Overall the GAI is an easy to use instrument, its scoring is simple, and it takes a short time to be completed. However, as this is the first and only study to investigate the validity and reliability of the GAI among older Iranian, more research is needed to confirm the findings. Future studies should test the properties of the scale and explore the correlates of anxiety in clinical samples of elderly of different populations, such as those who reside in the community or in nursing homes. Yet, the findings from current study could help gerontologist to use this questionnaire for screening anxiety among elderly populations. The GAI is based on DSM symptoms of anxiety, and thus it can help clinicians reach at an accurate diagnosis in clinical practice.

### Limitations

This study has some limitations. First we did not carry out cognitive interviews. Secondly we did not perform test-retest analysis in order to assess stability. Finally, we recruited our sample only from community while it seems that including samples from nursing home cares or gerontology departments in hospitals might further confirm that the questionnaire could be used in different settings.

## Conclusion

Overall the results suggest that the GAI-P has good psychometric properties and now can be used in clinical and research settings in order to measure social anxiety among elderly populations. Indeed measuring social anxiety is very relevant to improve good health in this population and perhaps reduce suffering from loneliness and social isolation. Further psychometric evaluation such as assessment of stability and responsiveness to change are recommended for the future studies.

## Data Availability

The datasets are available from the corresponding authors on request.

## Abbreviations

CFA: Confirmatory Factor Analysis
CFI: Comparative Fit Index
EFA: Exploratory Factor Analysis
GAI: Geriatric Anxiety Inventory
KMO: Kaiser–Meyer–Olkin
RMSEA: Root mean square error of approximation
SRMR: Standardized Root Mean Square Residual
TLI: Tucker-Lewis Index
WLSMV: Weighted Least Square Mean and Variance adjusted
WRMR: Weighted Root Mean Square Residual
